# Neonatal Mortality Disparities by Gestational Age in European Countries

**DOI:** 10.1001/jamanetworkopen.2024.24226

**Published:** 2024-08-07

**Authors:** Victor Sartorius, Marianne Philibert, Kari Klungsoyr, Jeannette Klimont, Katarzyna Szamotulska, Zeljka Drausnik, Petr Velebil, Laust Mortensen, Mika Gissler, Jeanne Fresson, Jan Nijhuis, Wei-Hong Zhang, Karin Källén, Tonia A. Rihs, Vlad Tica, Ruth Matthews, Lucy Smith, Jennifer Zeitlin

**Affiliations:** 1 CRESS, Obstetrical Perinatal and Paediatric Epidemiology Research Team, EPOPe, INSERM, INRA, Université Paris Cité, Paris, France; 2 Department of Neonatal Intensive Care, AP-HP, Hôpital Necker Enfants-Malades, Paris, France; 3 Division of Mental and Physical Health, Norwegian Institute of Public Health, Bergen, Norway; 4 Department of Global Public Health and Primary Care, University of Bergen, Bergen, Norway; 5 Unit Demography and Health, Directorate Social Statistics, Statistics Austria, Vienna, Austria; 6 Department of Epidemiology and Biostatistics, Institute of Mother and Child, Warsaw, Poland; 7 Division of Public Health, Croatian Institute of Public Health, Zagreb, Croatia; 8 Department of Obstetrics and Gynecology, Institute for the Care of Mother and Child, Prague, Czech Republic.; 9 Department of Obstetrics and Gynecology, Third Faculty of Medicine, Charles University, Prague, Czech Republic; 10 Section for Epidemiology, Department of Public Health, University of Copenhagen, Copenhagen, Denmark; 11 Department of Knowledge Brokers, Finnish Institute for Health and Welfare, Helsinki, Finland; 12 Region Stockholm, Academic Primary Health Care Centre, Stockholm, Sweden; 13 Karolinska Institutet, Department of Molecular Medicine and Surgery, Stockholm, Sweden; 14 Direction de la Recherche, des Etudes, de l’Evaluation et des Statistiques (Drees), Paris, France; 15 Department of Obstetrics and Gynecology, Maastricht University Medical Centre, MUMC+, Maastricht, the Netherlands; 16 International Centre for Reproductive Health, Department of Public Health and Primary Care, Faculty of Medicine and Health Sciences, Ghent University, Ghent, Belgium; 17 Swedish National Board of Health and Welfare, Department of Analysis, Stockholm, Sweden; 18 Federal Statistical Office (FSO), Neuchâtel, Switzerland; 19 Faculty of Medicine, East European Institute for Reproductive Health, Academy of Romanian Scientists, University ‘Ovidius’ Constanţa, Constanța, Romania; 20 Department of Population Health Sciences, College of Life Sciences, University of Leicester, Leicester, United Kingdom

## Abstract

**Importance:**

There are wide disparities in neonatal mortality rates (NMRs, deaths <28 days of life after live birth per 1000 live births) between countries in Europe, indicating potential for improvement. Comparing country-specific patterns of births and deaths with countries with low mortality rates can facilitate the development of effective intervention strategies.

**Objective:**

To investigate how these disparities are associated with the distribution of gestational age (GA) and GA-specific mortality rates.

**Design, Setting, and Participants:**

This was a cross-sectional study of all live births in 14 participating European countries using routine data compiled by the Euro-Peristat Network. Live births with a GA of 22 weeks or higher from 2015 to 2020 were included. Data were analyzed from May to October 2023.

**Exposures:**

GA at birth.

**Main Outcomes and Measures:**

The study investigated excess neonatal mortality, defined as a rate difference relative to the pooled rate in the 3 countries with the lowest NMRs (Norway, Sweden, and Finland; hereafter termed the top 3). The Kitagawa method was used to divide this excess into the proportion explained by the GA distribution of births and by GA-specific mortality rates. A sensitivity analysis was conducted among births 24 weeks’ GA or greater.

**Results:**

There were 35 094 neonatal deaths among 15 123 428 live births for an overall NMR of 2.32 per 1000. The pooled NMR in the top 3 was 1.44 per 1000 (1937 of 1 342 528). Excess neonatal mortality compared with the top 3 ranged from 0.17 per 1000 in the Czech Republic to 1.82 per 1000 in Romania. Excess deaths were predominantly concentrated among births less than 28 weeks’ GA (57.6% overall). Full-term births represented 22.7% of the excess deaths in Belgium, 17.8% in France, 40.6% in Romania and 17.3% in the United Kingdom. Heterogeneous patterns were observed when partitioning excess mortality into the proportion associated with the GA distribution vs GA-specific mortality. For example, these proportions were 9.2% and 90.8% in France, 58.4% and 41.6% in the United Kingdom, and 92.9% and 7.1% in Austria, respectively. These associations remained stable after removing births under 24 weeks’ GA in most, but not all, countries.

**Conclusions and Relevance:**

This cohort study of 14 European countries found wide NMR disparities with varying patterns by GA. This knowledge is important for developing effective strategies to reduce neonatal mortality.

## Key Points

**Question:**

Are disparities in neonatal mortality between European countries associated with differences in the gestational age distribution of births or with higher mortality at specific gestational ages?

**Findings:**

In this cross-sectional study of over 15 million live births from 14 European countries, heterogeneous patterns were observed across countries and gestational age groups in the association of these 2 components with excess mortality in comparison with the 3 countries with the lowest neonatal mortality rates (Sweden, Norway, and Finland).

**Meaning:**

Stratifying neonatal mortality rates by gestational age can provide country-specific insights to inform preventive strategies to reduce neonatal mortality.

## Teaser

This cross-sectional study evaluates whether disparities in neonatal mortality rates are associated with the distribution of gestational age and gestational age–specific mortality rates.

## Introduction

Neonatal mortality, deaths occurring in the first 28 days of life after live birth, accounts for up to 70% of infant deaths and 50% of mortality in children aged less than 5 years.^1^ There are significant disparities in neonatal mortality rates (NMRs) worldwide, ranging from 27.2 per 1000 live births in Sub-Saharan African to 2.3 per 1000 live births in Western Europe in 2017.^2^ Rates also differ between high-income European countries, with NMRs differing 2-fold between low mortality and high mortality countries in Europe.^3^ While NMRs have declined over time,^4^ these differences have not diminished and contribute to the health burden of these deaths, estimated at over 11 000 deaths a year in Western European countries.^5^ Furthermore, in recent years, some European and other high-income countries, including France and the US, have reported levelling off or even increasing neonatal mortality, after decreasing for decades.^6,7,8^

Preterm birth (birth before 37 weeks’ gestational age [GA]) is a principal cause of neonatal mortality, and up to 75% of neonatal deaths in high income countries occur among preterm babies.^9^ The risk of neonatal death varies by GA, from almost 1000 per 1000 live births for neonates born at 22 weeks’ GA to less than 1 per 1000 live births at 40 weeks.^10^ The preterm birth rate differs internationally, ranging from 4.1% to 8.2% in a study of 34 high income countries and regions,^11^ suggesting a varying proportion of births at risk of neonatal mortality. Such disparities in the distribution of births by GA could explain differences in the overall NMR. GA-specific mortality also varies widely, especially among babies born extremely preterm (<28 weeks’ GA), as shown in national cohorts and national neonatal register studies.^12,13,14,15,16,17^ Among 10 countries participating in the iNeo network, for instance, mortality before hospital discharge at 24 weeks’ GA ranged from 26% to 65%.^17^ This variation in mortality rates by GA may also contribute to variations in overall NMRs.

Understanding how the GA distribution of live births, as well as varying GA-specific mortality, contributes to differences in NMRs could enrich international comparisons and provide insights into effective preventive strategies. Public policy interventions to reduce preterm birth focus primarily on antenatal measures and broader population prevention,^18^ while measures to decrease GA-specific NMR target the organization and quality of obstetrical and neonatal care. Furthermore, actions targeting very preterm mortality may vary from those used to reduce neonatal deaths at full term.

Methods like decomposition and standardization are often used by demographers to quantify the compositional and rate effects of specific factors on mortality rate differences, but are not regularly applied in perinatal health surveillance or research. In this study, we examine NMRs in 14 European countries. Using the 3 countries with the lowest rates (Finland, Norway, and Sweden) as a reference, we aim to quantify excess mortality and to decompose the proportion of this excess mortality attributed to the GA distribution and to GA-specific mortality for each country.

## Methods

### Data Sources and Study Population

This study uses data from the Euro-Peristat Network, which collects data on perinatal indicators in Europe from routine population-based sources (ie, medical birth registers, civil registration, and hospital data systems).^3^ Data from January 2015 to December 2020 were collected using a standardized federated protocol. Participating data providers formatted data into a common data model and ran the same R scripts to produce aggregated data tables, which were compiled into a central database for analysis.^19^ The Euro-Peristat protocol collects aggregated, country-level, anonymous data for which ethics approval and the need for informed consent are not required by European data protection regulations.^19^ Data were collected in 2021 and 2022 and this analysis was conducted in 2023, following the Strengthening the Reporting of Observational Studies in Epidemiology (STROBE) reporting guideline for cross-sectional studies.

The study population included all singleton and multiple live births with a GA of 22 or more weeks or if GA was missing. We included countries with more than 200 000 live births during the 6-year study period to have a sufficiently large sample size for GA-specific analyses. Countries that could not provide data on neonatal deaths and/or GA at birth (defined as the final estimate in obstetrical records) were excluded from the analysis. Data for France were from 2015 to 2017 because neonatal death certificates were only available for years up to 2017. In Romania, data were only available from 2017 to 2020. Fourteen countries were included: Austria, Belgium, Croatia, the Czech Republic, Denmark, Finland, France, the Netherlands, Norway, Poland, Romania, Sweden, Switzerland, and the United Kingdom (UK). Data sources used in each country are listed in eTable 1 in Supplement 1.

### Missing Data

Overall, the number of births with missing GA was low; 0.8% for live births ending in a neonatal death and 1.3% for other live births (eTable 2 in Supplement 1). The extent of missing data varied between countries but was below 5% for all of them. Missing GA was imputed for each country based on the distribution of reported GA separately for neonatal deaths and for live births surviving to 28 completed days of life, which assumes that data are missing at random.

### Statistical Analysis

The NMR was calculated as the number of deaths from 0 to 27 completed days of life among live births born at 22 or more weeks’ GA per 1000 live births. Finland, Norway and Sweden had lower overall NMRs than the other European countries and are frequently cited as benchmarks for perinatal health.^20,21^ Therefore, we pooled their data to serve as the reference for comparison with the other countries (referred to subsequently as the top 3).

The rate difference in NMR compared with the top 3 was calculated for all other countries. The proportion of this rate difference explained by the GA distribution or GA-specific mortality was calculated using the Kitagawa decomposition,^22^ as follows: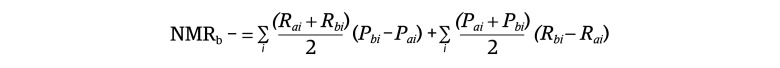.The Kitagawa formula uses information on the mortality rate (*R*) and the proportion (*P*) of live births for each GA (*i*) in the top 3 reference population (*a*) and the country to be compared (*b*). The first component of the equation computes the rate difference attributed to the GA distribution, while the second gives the rate difference due to GA-specific mortality. The number of excess deaths attributed to each component (the GA distribution vs the GA-specific mortality rate) can then be obtained by multiplying these rates by the total number of live births in each country and expressed as a percentage of the total number of excess deaths. These percentages range from 0% to 100% and sum to 100%. In some cases, when 1 of the components is associated with a lower mortality rate than the top 3, the percentage of excess deaths can be negative, leading to a percentage exceeding 100% for the other component. In these cases, values were truncated to 0% and 100%. For France and Romania, data were only available for 2015 to 2017 and 2017 to 2020, respectively, and comparisons were made with subsets of the pooled data from the top 3 for the corresponding periods.

Wide variations between countries have been shown in reporting among births at 22 and 23 weeks of GA, near the limits of viability.^23^ These differences have been linked to national policies of survival-focused care and also to differences in the reporting of births as live births or stillbirths.^24^ Therefore, we conducted a sensitivity analysis excluding live births less than 24 weeks’ GA.

Analyses were conducted using R version 4.2.2 (R Project for Statistical Computing).^25^ Data were analyzed from May to October 2023.

## Results

During the study period, 15 123 428 live births at 22 or more weeks’ GA were recorded in the 14 participating countries and 35 094 infants died in their first 28 days of life, giving an overall NMR of 2.32 per 1000 live births. The top 3 had the lowest NMRs, and the combined NMR for the top 3 was 1.44 per 1000 live births (1937 deaths for 1 342 528 live births). In the 11 other countries, NMRs ranged from 1.61 per 1000 live births (Czech Republic; 1086 deaths for 674 299 live births) to 3.26 per 1000 live births (Romania; 2759 deaths for 846 842 live births) with a median (IQR) rate of 2.22 (1.88-2.84) per 1000 live births (Table 1). Both the GA distribution and GA-specific mortality differed between countries, with the highest NMRs being more than twice the lowest NMRs in all GA groups (Table 2).

**Table 1.** Study Population, Live Births and Neonatal Deaths From 2015 to 2020, Euro-Peristat Database

Country	Individuals, No. (%)		NMR per 1000 live births
	Live births	Neonatal deaths	
All countries	15 123 428 (100.0)	35 094 (100.0)	2.32
Top 3	1 342 528 (8.9)	1937 (5.5)	1.44
Norway	340 518 (2.3)	475 (1.3)	1.39
Finland	299 796 (2.0)	425 (1.2)	1.42
Sweden	702 214 (4.6)	1037 (3.0)	1.48
Czech Republic	674 299 (4.5)	1086 (3.1)	1.61
Austria	509 397 (3.4)	880 (2.5)	1.73
Denmark	367 021 (2.4)	640 (1.8)	1.74
Switzerland	519 598 (3.4)	1054 (3.0)	2.03
United Kingdom	4 450 511 (29.4)	9471 (27.0)	2.13
Belgium	710 553 (4.7)	1575 (4.5)	2.22
France^a^	2 227 118 (14.7)	5629 (16.0)	2.53
Poland	2 265 054 (15.0)	6215 (17.7)	2.74
Croatia	221 274 (1.5)	651 (1.9)	2.94
The Netherlands	989 233 (6.5)	3197 (9.1)	3.23
Romania^b^	846 842 (5.6)	2759 (7.9)	3.26

Abbreviation: NMR, neonatal mortality rate.

^a^ Data for the 2015 to 2017 period only.

^b^ Data for the 2017 to 2020 period only.

**Table 2.** Neonatal Deaths and Live Births by Gestational Age (GA), per 1000 Live Births, 2015 to 2020, Euro-Peristat Database

Country	Rate per 1000 live births by GA group, completed wk						
	22-23	24-25	26-27	28-31	32-36	37-41	≥42
**Neonatal mortality rate**							
Finland, Norway, and Sweden	491.85	174.53	71.73	30.89	5.21	0.52	0.42
Czech Republic	681.22	301.91	106.40	29.14	4.11	0.41	0.32
Austria	695.52	212.26	86.81	27.93	3.96	0.43	1.34
Denmark	901.84	326.15	103.39	30.95	4.44	0.45	0.87
Switzerland	687.50	292.08	74.45	24.73	5.06	0.59	1.03
UK	668.37	226.55	87.88	29.60	5.20	0.65	0.57
Belgium	910.78	374.59	120.40	32.92	3.97	0.71	0.93
France^a^	1000.00	440.46	160.30	39.48	5.53	0.72	0.68
Poland	807.28	456.04	198.23	59.84	7.74	0.65	0.85
Croatia	875.82	525.42	167.58	78.26	7.74	0.67	0.00
The Netherlands	967.18	393.86	138.06	44,86	7.90	0.78	1.42
Romania^b^	666.67	472.31	291.44	64.00	9.07	1.34	3.30
**Live birth GA distribution**							
Finland, Norway, and Sweden	0.55	0.99	1.39	5.40	48.36	893.32	49.98
Czech Republic	0.34	1.16	1.71	7.33	60.91	900.93	27.61
Austria	0.66	1.25	1.74	7.17	63.93	922.33	2.93
Denmark	0.44	1.01	1.53	6.43	51.60	916.99	22.00
Switzerland	0.83	1.17	1.50	6.14	59.23	925.54	5.59
UK	0.70	1.35	1.97	7.98	65.85	900.44	21.70
Belgium	0.38	1.28	1.84	7.10	70.19	917.70	1.51
France^a^	0.44	1.17	1.86	6.85	60.38	920.67	8.63
Poland	0.53	1.13	1.60	6.58	62.24	921.17	6.74
Croatia	0.69	1.07	1.65	5.72	56.65	914.17	20.06
The Netherlands	1.05	1.22	1.84	6.80	56.15	920.01	12.83
Romania^b^	0.06	0.77	1.30	8.73	74.87	909.26	5.01

^a^ Data for the 2015 to 2017 period only.

^b^ Data for the 2017 to 2020 period only.

Results of the Kitagawa decomposition are presented in Table 3. The subdivision of excess mortality between the GA distribution and GA-specific mortality differed between countries. In many countries, the GA distribution was associated with a low proportion of the excess mortality (Belgium, 23.0%; Croatia, 16.6%; Denmark, 0.0%; the Czech Republic, 27.7%; France, 9.2%; Poland, 16.1%; and Romania, 0.0%), with higher GA-specific mortality associated with between 72.3% and 100% of excess mortality. Almost all the excess deaths in Austria were attributed to a higher proportion of births with lower GA (92.9%), whereas in the Netherlands, Switzerland, and the UK, the associations with the GA distribution and specific mortality were more balanced: 34.3% and 65.7%, 53.5% and 46.5%, and 58.4% and 41.6% in each country, respectively.

**Table 3.** Neonatal Mortality Rate (NMR) Differences for Each Country With Sweden, Norway, and Finland and the Kitagawa Decomposition Into the Differences Attributed to the Gestational Age (GA) Distribution and to GA-Specific NMRs Among Live Births at 22 Weeks or More and 24 Weeks or More of GA^a^

Country	NMR per 1000 live births	Difference from Finland, Norway, and Sweden	% of the Difference attributed to	
			GA distribution	GA-specific NMR
Live births ≥22 weeks of GA				
Finland, Norway, and Sweden	1.44	NA	NA	NA
Czech Republic	1.61	0.17	27.7	72.3
Austria	1.73	0.28	92.9	7.1
Denmark	1.74	0.30	0.0	100.0
Switzerland	2.03	0.59	53.5	46.5
UK	2.13	0.69	58.4	41.6
Belgium	2.22	0.77	23.0	77.0
France^b^	2.53	1.03	9.2	90.8
Poland	2.74	1.30	16.1	83.9
Croatia	2.94	1.50	16.6	83.4
The Netherlands	3.23	1.79	34.3	65.7
Romania^b^	3.26	1.82	0.0	100.0
Live births ≥24 weeks of GA				
Finland, Norway, and Sweden	1.17	NA	NA	NA
Czech Republic	1.38	0.21	87.8	12.2
Austria	1.27	0.10	100.0	0.0
Denmark	1.34	0.17	28.2	71.8
Switzerland	1.46	0.28	47.0	53.0
UK	1.66	0.49	64.5	35.5
Belgium	1.87	0.70	42.0	58.0
France^b^	2.08	0.88	21.1	78.9
Poland	2.31	1.14	19.8	80.2
Croatia	2.34	1.16	12.7	87.3
The Netherlands	2.22	1.05	21.9	78.1
Romania^c^	3.22	2.04	11.3	88.7

Abbreviation: NA, not applicable.

^a^ Percentages are truncated to a range from 0% to 100%.

^b^ Data for the 2015 to 2017 period only. Comparison was made with the NMR of the top 3 over the same period: 1.50 and 1.20 for live births 22 or more and 24 or more weeks’ GA respectively.

^c^ Data for the 2017 to 2020 period only. Comparison was made with the NMR of the top 3 over the same period: 1.43 and 1.18 for live births 22 or more and 24 or more weeks’ GA respectively.

When live births before 24 weeks’ GA were excluded from the analysis, the difference between the pooled NMR in the top 3 and the NMR in the 11 other countries decreased from 0.97 per 1000 live births to 0.78 per 1000 live births. The excess mortality attributed to the GA distribution was higher in countries like Belgium (increased from 23.0% to 42.0%) and in the UK (increased from 58.4% to 64.5%), but it was also lower in others like the Netherlands (decreased from 34.3% to 21.9%). Overall, the main component of the excess mortality remained the same, except for the Czech Republic, where the GA distribution accounted for 27.7% of excess deaths when using all included GAs but became the main component (87.8%) after excluding births at less than 24 weeks’ GA.

The number of neonatal deaths considered in excess by GA group in relation to the top 3 is displayed in Table 4. Overall, NMR disparities accounted for a large number of neonatal deaths, estimated at 13 159 (37.5% of all deaths for 9 countries 2015-2020, France 2015-2017, and Romania 2017-2020), of which 83.6% were before full term and 16.4% were at full term. Over half of the excess neonatal deaths were concentrated among extremely preterm births at less than 28 weeks’ GA in all countries (57.6% overall), except for Romania, where they composed about one-quarter. Full-term births represented 22.7% of the excess deaths in Belgium, 17.8% in France, 40.6% in Romania and 17.3% in the United Kingdom.

**Table 4.** Differences in the Number of Neonatal Deaths Expected if the Gestational Age (GA) Distribution and GA-Specific Mortality Rates Were the Same as Sweden, Norway, and Finland

Country	Number of neonatal deaths in excess by GA group in completed weeks, No. (% of all deaths in excess)							
	22-23	24-25	26-27	28-31	32-36	37-41	≥ 42	All, No.
Czech Republic	−26 (−22.8)	120 (106.1)	56 (49.2)	31 (27.8)	−1 (−0.7)	−59 (−52.7)	−8 (−7.1)	113
Austria	96 (65.8)	47 (32.2)	26 (17.8)	17 (11.6)	1 (0.7)	−32 (−21.9)	−9 (−6.2)	146
Denmark	48 (43.6)	57 (51.8)	21 (19.1)	12 (10.9)	−8 (−7.3)	−19 (−17.3)	−1 (−0.9)	110
Switzerland	157 (51.4)	87 (28.4)	6 (2.0)	−8 (−2.5)	25 (8.2)	46 (15.0)	−8 (−2.5)	305
UK	892 (29.2)	590 (19.3)	327 (10.7)	308 (10.1)	405 (13.3)	566 (18.6)	−38 (−1.2)	3050
Belgium	53 (9.7)	219 (39.8)	86 (15.7)	47 (8.6)	19 (3.5)	139 (25.3)	−14 (−2.5)	550
France^a^	333 (14.5)	750 (32.7)	396 (17.3)	251 (10.9)	153 (6.7)	441 (19.2)	−32 (−1.4)	2292
Poland	365 (12.4)	774 (26.2)	492 (16.7)	514 (17.4)	521 (17.7)	315 (10.7)	−34 (−1.2)	2947
Croatia	74 (22.4)	86 (25.8)	39 (11.7)	62 (18.7)	41 (12.4)	34 (10.3)	−5 (−1.4)	331
The Netherlands	735 (41.5)	303 (17.1)	152 (8.6)	137 (7.7)	190 (10.7)	255 (14.4)	−3 (−0.1)	1769
Romania^b^	−182 (−11.8)	147 (9.5)	247 (16.0)	338 (21.9)	368 (23.8)	634 (41.0)	−6 (−0.4)	1546

^a^ Data for the 2015 to 2017 period only.

^b^ Data for the 2017 to 2020 period only.

The fraction of the difference in the number of neonatal deaths compared with the top 3 attributed to the composition of GA distribution and GA-specific mortality rate is presented by GA in the Figure for the 4 countries with the highest number of births during the study period and in eFigure 1 and eFigure 2 in Supplement 1 for the others. In the Netherlands, the mortality excess at 22 weeks’ GA was mainly associated with a higher number of live births at that GA, whereas GA-specific mortality was the predominant component from 23 weeks’ GA. In Poland, 90.4% of excess deaths were preterm births less than 37 weeks’ GA, and most of them were due to GA-specific mortality. In the UK, the 2 components explained the excess of deaths before 28 weeks’ GA, whereas the distribution component and the specific mortality component were more pronounced from 28 to 36 weeks’ GA and from 37 weeks’ GA, respectively. The total number of deaths is equal to the difference between the 2 bars when the association is negative, as occurs at the extremes; for example fewer deaths at 22 and 23 weeks in France and Poland or at 42 weeks in the Netherlands.

**Figure.**
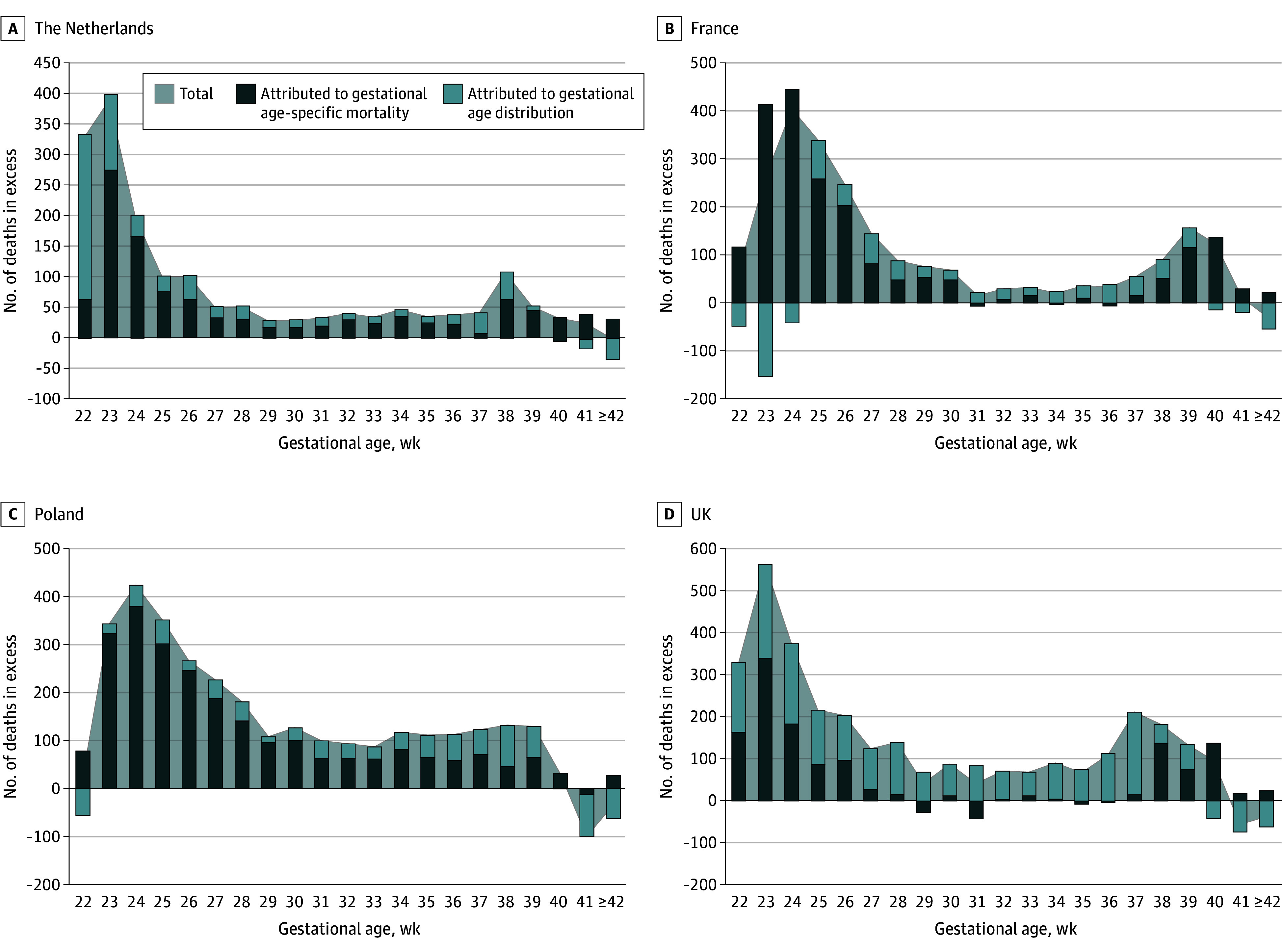
Number of Neonatal Deaths Considered in Excess by Gestational Age in Comparison With Sweden, Norway, and Finland The shaded area indicates the total number of neonatal deaths considered in excess, in each country and at each gestational age compared with Sweden, Norway, and Finland. The total number of excess deaths is the sum of the number of excess deaths attributed to gestational age distribution and the number of excess deaths attributed to gestational age-specific mortality, represented respectively by the light blue and dark blue bars. If a bar indicates a negative value, it means that the proportion of births at that gestational age is favorable compared with Sweden, Norway, and Finland (light blue bar), or that the mortality rate at that gestational age is favorable compared with Sweden, Norway, and Finland (dark blue bar). This figure displays the 4 countries with the highest number of births during the study period. The other countries included in the study can be found in eFigure 1 and eFigure 2 in Supplement 1.

## Discussion

This study provides novel insights into international differences in rates of neonatal mortality, revealing significant variations in the association of the distribution of GA among live births vs GA-specific mortality rates to NMR disparities in European countries. Our population-based analysis of all live births over a 6-year period revealed significant excess mortality when 11 European countries were compared with the top 3, totaling about 40% of their neonatal deaths. This excess was concentrated in the very preterm period and was also observed at full term, although the GA pattern of the mortality excess differed. The GA distribution did not explain a high proportion of excess mortality in the majority of countries. For example, higher rates of mortality at specific GAs were associated with all of the neonatal mortality gap in Denmark and almost all in France (90.8%). In contrast, Austria had GA-specific NMRs that were close to the pooled rate in the top 3 countries, but preterm birth rates were higher. In the Netherlands, Switzerland, and the UK, both higher preterm birth rates and higher mortality rates at specific GAs were associated with the gap (34.3% and 65.7% in the Netherlands, 53.5% and 46.5% in Switzerland, 58.4% and 41.6% in the UK, respectively). By highlighting differences in preterm birth rates and in mortality risks at specific GAs, these results can provide guidance for investigations into the causes of avoidable neonatal deaths and for national public health policies to reduce neonatal mortality.

We have shown that the Kitagawa decomposition method, which is easy to implement, can add value to international benchmarking exercises exploring inequalities in perinatal health. This algorithm has already been employed to analyze infant mortality within the US and to compare the US with Europe, although larger GA groups were used.^26^ In these studies, preterm birth was responsible for 51% of the excess of infant mortality in the southern states in comparison with other states,^27^ and for 39% of excess mortality of the US in comparison with Sweden.^28^ These previous results underscored the substantial yet varying role of GA distribution and support our study’s call for more systematic consideration of GA in comparisons of neonatal and infant mortality rates.

There are multiple, coexisting explanations for differences in NMRs and decomposing these differences by GA may shed light on those of most importance. On a global scale, neonatal mortality and preterm birth rates are highly correlated with measures of countries’ socioeconomic development.^1,29^ All the countries included in the study are classified as high-income countries and have accessible, high-quality national health care systems.^30^ Still, poverty remains a major risk factor for adverse perinatal outcomes.^31^ In the UK, for example, socioeconomic deprivation was estimated to be responsible for 24% of neonatal deaths.^32^ In this study, most of the excess deaths associated with deprivation were due to preterm births. Socioeconomic deprivation has also been associated with higher rates of GA-specific mortality,^33^ and poor infant outcomes have been shown to be more common in hospitals serving minority and disadvantaged populations.^34^

A number of demographic factors and global health determinants have been independently associated with the incidence of preterm birth, and may account for differences between countries. Some of them reflect characteristics of the childbearing population, such as younger and older maternal age, high parity, and ethnicity.^35^ Others may be amenable to preventive measures, including exposure to risk factors like smoking and air pollution and maternal health conditions such as obesity, diabetes, and hypertension.^36,37^ Multiple births are also linked to higher preterm birth rates, and their incidence differs between European countries.^38^ Multiple birth rates reflect the distribution of maternal age, but also policies and procedures related to subfertility and, in particular, adoption of single embryo transfer policies.^39^

Neonatal mortality also varies depending on the organization of perinatal health care pathways and clinical practices. There are considerable differences between European countries in antenatal care and screening.^40^ These may affect the identification of medical complications and referral to appropriate health care facilities, which may impact both the probability of preterm or early term birth and GA-specific mortality rates. Some health care factors are relevant to neonatal mortality for specific risk groups. For instance, being born in a specialized facility favors survival for babies born extremely preterm or with some congenital anomalies (certain heart anomalies, congenital diaphragmatic hernias, or gastroschisis).^41,42,43^ Prenatal screening also affects survival through fetal intervention techniques, such as laser therapy for twin-to-twin transfusion syndrome, or balloon occlusion of the trachea for congenital diaphragmatic hernia.^44,45^ Finally, screening policies affect detection of lethal congenital anomalies, and neonatal mortality will decrease if decisions are made to terminate these pregnancies.^46^ Policies regarding late termination of pregnancy differ between countries in Europe, being highly restricted from 22 weeks’ GA in Denmark, Poland, Norway, and Sweden, with no GA limitations in Belgium and France.^47^

Other clinical practices differ between countries which affect both the GA distribution and mortality rate. These include the administration of antenatal steroids, which increases the survival of very preterm babies,^48^ and the management of postterm pregnancies, which involves labor induction and shifts in GA distribution. Of note, the subgroup of 42 or more weeks’ GA was the only one where mortality was higher in the top 3 than in many other countries, primarily due to a higher proportion of postterm births. The Nordic countries have higher rates of postterm births than other countries,^49^ and this has been an area of changing practice and debate in these countries.^50^ Advances in neonatal care such as surfactant administration, improved ventilation techniques, and nutrition management, among others, have reduced mortality among extremely preterm infants,^2,51,52^ while procedures such as therapeutic hypothermia in hypoxic-ischemic encephalopathy have reduced mortality among term births.^53^ Differences in the use of evidence-based care should be explored to explain variations in mortality rates.

Another cause of disparities is variation in the active management of babies born extremely preterm.^54^ These practice differences are most pronounced as GA approaches the limits of viability. Further, live births provided with survival-focused care may be more likely to be recorded in data systems leading to a higher proportion of live births as well as lower GA-specific mortality.^55^ Both active management and recording differences are likely to explain the significant differences we observed at 22 and 23 weeks’ GA^56^ and the impact of excluding these GAs from the analysis. In some countries, the positive association of these GA groups with a lower excess mortality is very likely due to underrecording of these births. This result suggests that overall neonatal mortality statistics may be more comparable when presented for births 24 weeks’ GA and over. This threshold is currently used by Euro-Peristat for stillbirth comparisons.^23^

### Strengths and Limitations

Strengths of this study include the large sample sizes as well as population-level data collected with a standardized protocol that allowed us to undertake a detailed analysis, with the calculation and the decomposition of the number of deaths in excess at each GA. Limitations include the absence of other population or health care data to allow further exploration of these differences. Comparative national-level analyses using individual data in Europe are complicated by strict laws on international transfer of data, but federated analysis systems that expand on protocols, such as that used by Euro-Peristat in this project, could integrate other key variables, such as maternal age, body mass index, and socioeconomic status, which are already included in Euro-Peristat’s aggregated outputs. Other limitations include missing data on GA which were observed in 10 of the 14 countries and were higher among neonatal deaths than among live births. However, overall proportions were low in most countries and we addressed this issue by imputing the missing data based on the existing distribution. Finally, Finland, Norway, and Sweden had the lowest NMRs in our study, and were selected as reference countries for this reason. However, the number of excess deaths calculated by country is only a theoretical estimate of potential progress compared with the top 3.

## Conclusions

Our cohort study of 14 European countries found significant differences between countries in how the GA distribution vs mortality rates at different GA was associated with excess neonatal mortality in 11 countries compared with the 3 European countries with lowest mortality. These results provide wide-ranging insights into inequalities seen in Europe and can guide the focus of public health actions toward reducing preterm birth, GA-specific mortality, or both. This method also enabled the identification of GA groups where excess mortality was most pronounced, which is key in guiding preventive measures.

## Notes

Supplement 1. **eTable 1.** Data Sources Used for the Euro-Peristat Network in the 14 Participating Countries **eTable 2. **Number of Cases (%) With Missing Gestational Age at Birth **eFigure 1. **Number of Neonatal Deaths Considered in Excess by Gestational Age in Comparison With the TOP3 (Sweden, Norway and Finland) **eFigure 2. **Number of Neonatal Deaths Considered in Excess by Gestational Age in Comparison With the TOP3 (Sweden, Norway and Finland)

Supplement 2.
Nonauthor Collaborators

Supplement 3.
Data Sharing Statement
