# Supplementary material for: Neonatal Mortality Disparities by Gestational Age in European Countries
Source: JAMA Netw Open. 2024 Aug 7;7(8):e2424226. doi: 10.1001/jamanetworkopen.2024.24226 (PMC11307138; doi:10.1001/jamanetworkopen.2024.24226)
Supplement: Supplement 1. — eTable 1. Data Sources Used for the Euro-Peristat Network in the 14 Participating Countries eTable 2. Number of Cases (%) With Missing Gestational Age at Birth eFigure 1. Number of Neonatal Deaths Considered in Excess by Gestational Age in Comparison With the TOP3 (Sweden, Norway and Finland) eFigure 2. Number of Neonatal Deaths Considered in Excess by Gestational Age in Comparison With the TOP3 (Sweden, Norway and Finland) [file jamanetwopen-e2424226-s001.pdf]

Supplemental Online Content

Sartorius V, Philibert M, Klunsoyr K, et al.Neonatal mortality disparities by gestational age in European countries. *JAMA Netw Open*. 2024;7(8):e2424226. doi:10.1001/jamanetworkopen.2024.24226

eTable 1. Data Sources Used for the Euro-Peristat Network in the 14 Participating Countries

eTable 2. Number of Cases (%) With Missing Gestational Age at Birth

eFigure 1. Number of Neonatal Deaths Considered in Excess by Gestational Age in Comparison With the TOP3 (Sweden, Norway and Finland)

eFigure 2. Number of Neonatal Deaths Considered in Excess by Gestational Age in Comparison With the TOP3 (Sweden, Norway and Finland)

This supplemental material has been provided by the authors to give readers additional information about their work.

© 2024 Sartorius V et al. *JAMA Network Open.*

eTable 1. Data Sources Used for the Euro-Peristat Network in the 14 Participating Countries

Country	Data sources
Austria	* Birth statistics (Statistics Austria)<br>* Cause of death statistics (Statistics Austria)
Belgium	* Vital Statistics, Statistics Belgium (Statbel)
Croatia	* Croatian Medical Birth Database (Croatian Public Health Institute)<br>* Croatian Mortality Database (Croatian Central Bureau of Statistics)
Czech Republic	* Institute of Health Statistics and Information of the Czech Republic (national birth register (mothers and newborns) collecting individual perinatal data.)
Denmark	* Medical birth register (The Danish Health Data Authority, Danish Ministry of Health)<br>* National patient register (The Danish Health Data Authority, Danish Ministry of Health)<br>* Danish causes of death register (The Danish Health Data Authority, Danish Ministry of Health)<br>* The Centralized Civil Register
Finland	* Medical Birth Register (Finnish Institute for Health Welfare) linked with Central Population Register (Digital and Population Data Services Agency) and Cause of Death Register (Statistics Finland)
France	* French National Health Data System (SNDS). Neonatal death certificates (CépiDC, Inserm) linked with hospital discharge data (PMSI, ATIH: Technical agency of hospitalization information).<br>*Civil birth register
The Netherlands	* Perined (The Netherlands Perinatal Registry)
Norway	* Medical Birth Register of Norway (The Norwegian Institute of Public Health)
Poland	* Central Statistical Office<br>* Ministry of Health
Romania	* National Institute for Public Health Romania
Sweden	* Medical Birth Register (The National Board of Health and Welfare)
Switzerland	* BEVNAT, statistics of natural population change - vital statistics (Swiss federal Statistical Office)
UK	* MBRRACE UK perinatal mortality surveillance (University of Oxford and University of Leicester)

© 2024 Sartorius V et al. *JAMA Network Open.*

eTable 2. Number of Cases (%) With Missing Gestational Age at Birth

Country	Neonatal deaths, No. (%)	Other live births, No. (%)
Austria	0 (0.0%)	0 (0.0%)
Belgium	47 (3.0%)	9 465 (1.3%)
Croatia	0 (0.0%)	4 193 (1.9%)
Czech Republic	43 (4.0%)	15 382 (2.3%)
Denmark	2 (0.3%)	568 (0.2%)
Finland	0 (0.0%)	453 (0.1%)
France	80 (1.4%)	9 465 (1.3%)
The Netherlands	56 (1.8%)	0 (0.0%)
Norway	0 (0.0%)	0 (0.0%)
Poland	19 (0.3%)	1 919 (<0.1%)
Romania	0 (0.0%)	0 (0.0%)
Sweden	7 (0.7%)	108 (<0.1%)
Switzerland	0 (0.0%)	0 (0.0%)
UK	17 (<0.1%)	158 459 (3.6%)
All countries	271 (0.8%)	200 012 (1.3%)

© 2024 Sartorius V et al. *JAMA Network Open.*

eFigure 1. Number of Neonatal Deaths Considered in Excess by Gestational Age in Comparison With the TOP3 (Sweden, Norway and Finland)

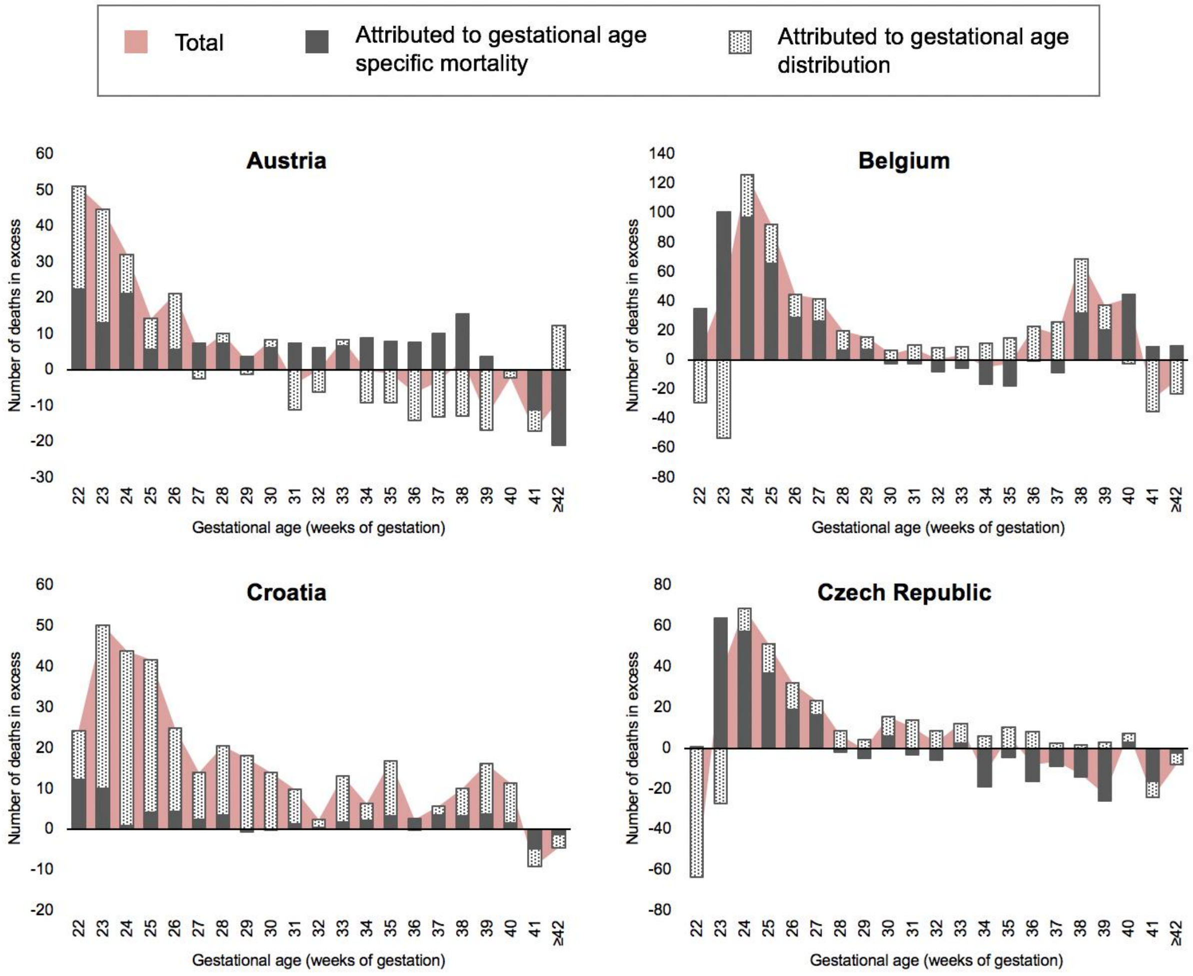

The orange area indicates the total number of neonatal deaths considered in excess, in each country and at each gestational age compared to the TOP3. The total number of excess deaths is the sum of the number of excess deaths attributed to gestational age distribution and the number of excess deaths attributed to gestational age-specific mortality, represented respectively by the dashed and gray bars. If a bar indicates a negative value, it means that the proportion of births at that gestational age is favorable compared to the TOP3 (dashed bar), or that the mortality rate at that gestational age is favorable compared to the TOP3 (grey bar).

© 2024 Sartorius V et al. *JAMA Network Open.*

eFigure 2. Number of Neonatal Deaths Considered in Excess by Gestational Age in Comparison With the TOP3 (Sweden, Norway and Finland)

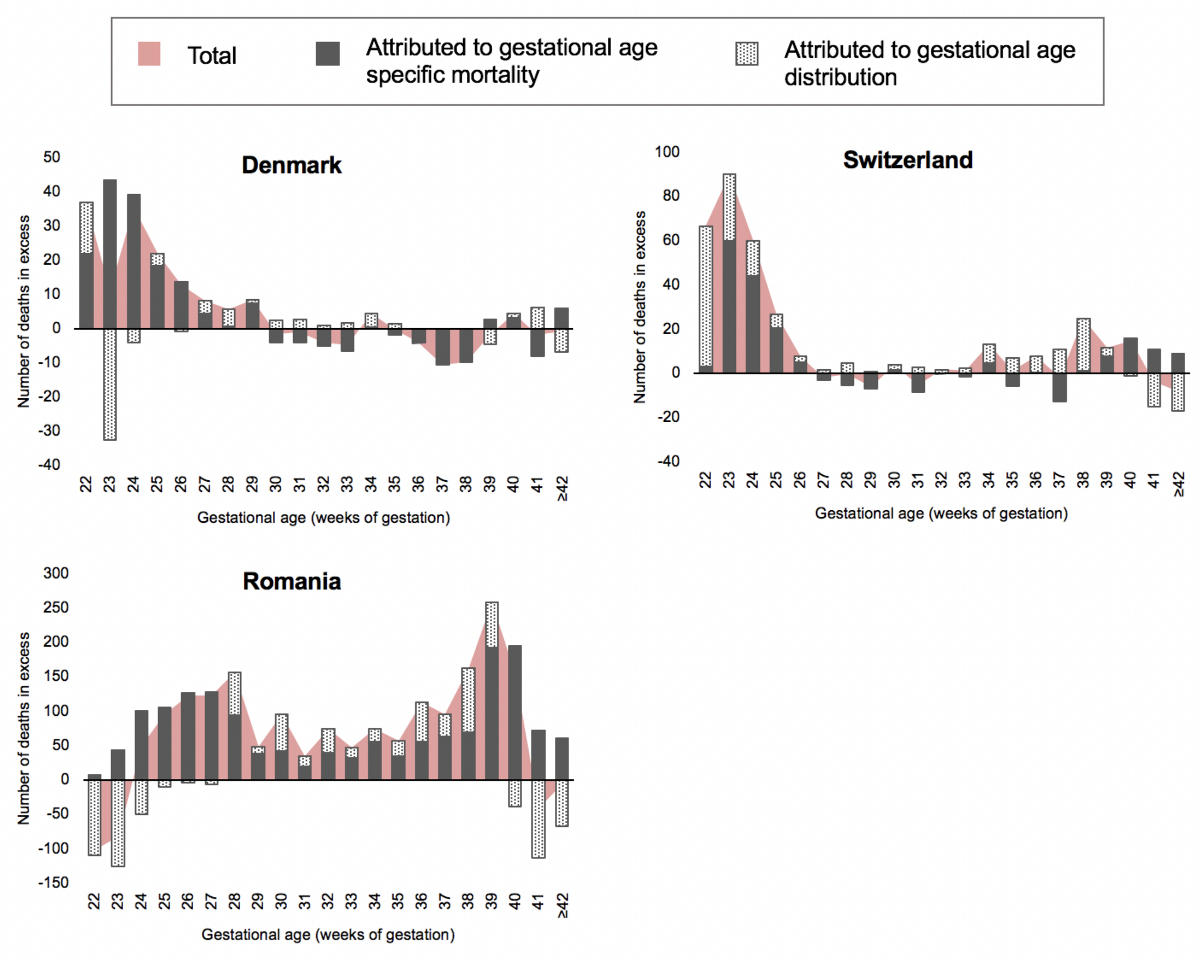

The orange area indicates the total number of neonatal deaths considered in excess, in each country and at each gestational age compared to the TOP3. The total number of excess deaths is the sum of the number of excess deaths attributed to gestational age distribution and the number of excess deaths attributed to gestational age-specific mortality, represented respectively by the dashed and gray bars. If a bar indicates a negative value, it means that the proportion of births at that gestational age is favorable compared to the TOP3 (dashed bar), or that the mortality rate at that gestational age is favorable compared to the TOP3 (grey bar).

© 2024 Sartorius V et al. *JAMA Network Open.*
