# Supplementary material for: Neonatal Mortality Disparities by Gestational Age in European Countries
Source: JAMA Netw Open. 2024 Aug 7;7(8):e2424226. doi: 10.1001/jamanetworkopen.2024.24226 (PMC11307138; doi:10.1001/jamanetworkopen.2024.24226)
Supplement: Supplement 2. — Nonauthor Collaborators [file jamanetwopen-e2424226-s002.pdf]

Supplementary Online Material: Nonauthor Collaborators
*First name, last name, and suffix (if applicable) are required and will appear in PubMed.

*Group Name(s): Euro-Peristat Network							
***First Name and Middle Initial(s)**	***Last Name**	***Suffix (eg, Jr, III)**	Academic Degrees	Institution	Location (city, state/province, country)	Role or Contribution, eg, chair, principal investigator	Group (if more than 1 Group listed in the byline) and/or Subgroup (eg, Steering Committee)
Alex	Farr			Department of Obstetrics and Gynecology, Medical University of Vienna, Vienna	AUSTRIA		
Sophie	Alexander			Université Libre de Bruxelles, School of Public Health, Epidemiology, Biostatistics and Clinical Research Centre	BELGIUM		
Judith	Racapé			Research Center in epidemiology, biostatistic and clinical research - School of Public Health	BELGIUM		
Gisèle	Vandervelpen			Statbel	BELGIUM		
Vasos	Coutellas			Health Monitoring Unit, Ministry of Health	CYPRUS		
Theopisti	Kyprianou			Health Monitoring Unit, Ministry of Health	CYPRUS		
Jitka	Jirova			Institute of Health Information and Statistics of the Czech Republic	CZECHIA		
Luule	Sakkeus			Estonian Institute for Population Studies, Tallinn University	ESTONIA		
Liili	Abuladze			Estonian Institute for Population Studies, Tallinn University	ESTONIA		
Béatrice	Blondel			INSERM, EPOPé	FRANCE		
Annick	Vilain			DREES	FRANCE		
Mélanie	Durox			INSERM, EPOPé	FRANCE		
Guenther	Heller			Institute for quality assurance and transparency in healthcare IQTIG	GERMANY		
István	Sziller			National Directory for Hospital Management, Budapest, Hungary	HUNGARY		
Johanna	Gunnarsdóttir			Landspitali University Hospital	ICELAND		

Supplementary Online Material: Nonauthor Collaborators
*First name, last name, and suffix (if applicable) are required and will appear in PubMed.

***First Name and Middle Initial(s)**	***Last Name**	***Suffix (eg, Jr, III)**	Academic Degrees	Institution	Location (city, state/province, country)	Role or Contribution, eg, chair, principal investigator	Group (if more than 1 Group listed in the byline) and/or Subgroup (eg, Steering Committee)
Helga	Sól Ólafsdóttir			Landspitali University Hospital	ICELAND		
Izabela	Sikora			The National Perinatal Reporting System, Health Pricing Office, Dublin	IRELAND		
Sinead	O'Hara			Healthcare Pricing Office (HPO), National Finance Division, HSE, Dublin	IRELAND		
Karen	Kearns			Healthcare Pricing Office (HPO), National Finance Division, HSE, Dublin	IRELAND		
Marina	Cuttini			Ospedale Pediatrico Bambino Gesù, Unit of Epidemiology	ITALY		
Marzia	Loghi			Central Directorate for Socio-demographic and Environmental Statistics, Italian National Institute for Statistics-ISTAT	ITALY		
Rosaria	Boldrini			Health information system and statistics, Ministry of health, Rome	ITALY		
Marilena	Pappagal			Italian National Institute for Statistics-ISTAT	ITALY		
Stefano	Marchetti			Italian National Institute for Statistics-ISTAT	ITALY		
Serena	Donati			National center for Disease Prevention and Health promotion - Istituto Superiore di Sanità, Italian Ministry of Health (ISS)-Rome	ITALY		
Janis	Misins			The Centre for Disease Prevention and Control of Latvia	LATVIA		
Irisa	Zile-Velika			The Centre for Disease Prevention and Control of Latvia	LATVIA		
Jelena	Isakova			Institute of Hygiene, Health Information Centre	LITHUANIA		

Supplementary Online Material: Nonauthor Collaborators
*First name, last name, and suffix (if applicable) are required and will appear in PubMed.

***First Name and Middle Initial(s)**	***Last Name**	***Suffix (eg, Jr, III)**	Academic Degrees	Institution	Location (city, state/province, country)	Role or Contribution, eg, chair, principal investigator	Group (if more than 1 Group listed in the byline) and/or Subgroup (eg, Steering Committee)
Aline	Lecomte			Department of Population Health, Luxembourg Institute of Health	LUXEMBOURG		
Jessica	Pastore			Department of Population Health, Luxembourg Institute of Health	LUXEMBOURG		
Daniel	Álvarez			Ministry of Health and Social Security, Health Directorate, Epidemiology and Statistics Unit	LUXEMBOURG		
Miriam	Gatt			National Obstetrics Information System, Directorate for Health Information and Research	MALTA		
PW	Achterberg			National Institute for Public Health and the Environment, Bilthoven	NETHERLANDS		
Lisa	Broeders			Perined	NETHERLANDS		
Rupali	Akerkar			Norwegian Institute of Public Health	NORWAY		
Hilde	Engjom			Division of Mental and Physical Health, Norwegian Institute of Public Health, Bergen	NORWAY		
Ewa	Mierzejewska			Institute of Mother and Child, Department of Epidemiology and Biostatistics, Warsaw	POLAND		
Henrique	Barros			University of Porto Medical School, Department of Clinical Epidemiology, Predictive Medicine and Public Health; Institute of Public Health	PORTUGAL		
Lucian	Puscasiu			East European Institute for Reproductive Health, University of Medicine, Pharmacy, Science and Technology “George Emil Palade” Tirgu-Mures	ROMANIA		

Supplementary Online Material: Nonauthor Collaborators
*First name, last name, and suffix (if applicable) are required and will appear in PubMed.

***First Name and Middle Initial(s)**	***Last Name**	***Suffix (eg, Jr, III)**	Academic Degrees	Institution	Location (city, state/province, country)	Role or Contribution, eg, chair, principal investigator	Group (if more than 1 Group listed in the byline) and/or Subgroup (eg, Steering Committee)
Mihaela-Alexandra	Budianu			Obstetrics and Gynaecology Clinic, University of Medicine and Pharmacy Târgu Mureş, Romania	ROMANIA		
Alexandra	Cucu			National Centre for Health Promotion and Evaluation- National Institute of Public Health	ROMANIA		
Vlad	Tica			East European Institute for Reproductive Health, Faculty of Medicine, University "Ovidius" Constanţa	ROMANIA		
Miha	Lučovnik			Department of Obstetrics and Gynecology, university medical center, Ljubljana	SLOVENIA		
Ivan	Verdenik			Department of Obstetrics and Gynecology, university medical center, Ljubljana	SLOVENIA		
Maria	Fernandez Elorriaga			Nursing department. School of Medicine - Autonomous University of Madrid	SPAIN		
Oscar	Zurriaga			1)Preventive Medicine and Public Health Departament, University of Valencia, Spain 2)FISABIO, Rare Diseases Research Area and Mixed Unit FISABIO-UVEG	SPAIN		
Adela	Recio Alcaide			Universidad de Alcala, Spain	SPAIN		
Anastasia	Nyman			The National Board of Health and Welfare, Department of Evaluation and Analysis, Epidemiology and Methodological Support Unit	SWEDEN		

Supplementary Online Material: Nonauthor Collaborators
*First name, last name, and suffix (if applicable) are required and will appear in PubMed.

***First Name and Middle Initial(s)**	***Last Name**	***Suffix (eg, Jr, III)**	Academic Degrees	Institution	Location (city, state/province, country)	Role or Contribution, eg, chair, principal investigator	Group (if more than 1 Group listed in the byline) and/or Subgroup (eg, Steering Committee)
Alison	Macfarlane			Maternal and Child Health and Care Research Centre, City University London	UK		
Sonya	Scott			Public Health Scotland	UK		
Kirsten	Monteath			Public Health Scotland	UK		
Siobhan	Morgan			Hospital Information Branch-Department of Health - Stormont Estate- Belfast	UK		
Craig	Thomas			Welsh Government	UK		
Sinead	Magill			Northern Ireland Maternal And Child Health (NIMACH)	UK		
Greg	Ceely			Office for National Statistics	UK		
