# Supplementary material for: Neonatal Mortality Disparities by Gestational Age in European Countries
Source: JAMA Netw Open. 2024 Aug 7;7(8):e2424226. doi: 10.1001/jamanetworkopen.2024.24226 (PMC11307138; doi:10.1001/jamanetworkopen.2024.24226)
Supplement: Supplement 3. — Data Sharing Statement [file jamanetwopen-e2424226-s003.pdf]

Data Sharing Statement

Sartorius. Neonatal Mortality Disparities by Gestational Age in European Countries. *JAMA Netw Open*. Published August 07, 2024. doi:10.1001/jamanetworkopen.2024.24226

Data
Data available: No
